# Massage-Related Changes in Cortical Activity and Cerebral Oxygenation in Healthy Term Infants: An Exploratory EEG-fNIRS Study with Sex-Specific Observations

**DOI:** 10.3390/neurolint18070131

**Published:** 2026-07-09

**Authors:** Rocío Llamas-Ramos, Jorge Juan Alvarado-Omenat, Daniel García-García, Ismael Sanz-Esteban, Juan Luis Sánchez-González, J. Ignacio Serrano, Inés Llamas-Ramos

**Affiliations:** 1 Department of Nursing and Physiotherapy, Universidad de Salamanca, 37007 Salamanca, Spaininesllamas@usal.es (I.L.-R.); 2 Instituto de Investigación Biomédica de Salamanca (IBSAL), 37007 Salamanca, Spain; 3 FisioSport Salamanca, 37008 Salamanca, Spain; 4 Neurosciences and Physical Therapy Research Group, Department of Physiotherapy, Faculty of Sport Sciences, Universidad Europea de Madrid, 28670 Madrid, Spain; 5 Computational Modeling of Intelligence (COMODIN) Group, Center for Automation and Robotics, CSIC-UPM, Ctra. Campo Real km 0.200, 28500 Arganda Del Rey, Spain; 6 University Hospital of Salamanca, 37007 Salamanca, Spain

**Keywords:** newborn, development, massage, EEG, fNIRS

## Abstract

Background: Central nervous system development is a rapid and highly plastic process during the first years of life. Tactile stimuli have been shown to induce cortical changes, but potential sex-related differences remain unexplored. This study aimed to investigate sex-specific differences in cortical activity and cerebral oxygenation in response to tactile stimulation via body massage. Methods: Four healthy full-term infants (two females and two males), all aged 11 weeks, were included in this prospective exploratory study. Each infant received a standardized 5 min massage protocol. Cortical activity and cerebral oxygenation were assessed using an 8-channel electroencephalogram (EEG) and functional near-infrared spectroscopy (fNIRS) before, during, and after the intervention, with a 5 min pre-intervention resting period used as the baseline. Results: EEG analysis focused on a single spectral band (4 Hz–30 Hz). This range was selected to capture the main cortical oscillations in infants, including theta, alpha, and beta activity, while delta activity below 4 Hz was partially excluded to reduce movement and physiological artifacts. Standard infant EEG bands were considered when defining this range. Data shows for the female subject an average PSD of −6.726 (± −4.075), and for the male subject, −12.594 (± −10.741). Although babies are of the same gestational age, they exhibited distinct basal cortical activity, which prevented comparisons from being made. Nevertheless, massage induced similar activity patterns in all subjects with increased cortical electrical activity in the left parietal region relative to baseline. fNIRS data showed that comparable HbO concentration patterns between participants were observed only during the second minute of recording. Relative to baseline, pre-intervention HbO responses displayed an opposite distribution, and the effects of the intervention differed by sex. The female participant exhibited a slight reduction in activation in the right hemisphere accompanied by a modest increase in the most ventral region of the left hemisphere. Conversely, the male participant showed an inverse response pattern, characterized by a marked increase in right hemispheric activation and a pronounced decrease in the left hemisphere during the intervention period. Conclusions: These preliminary observations suggest the presence of early variations in cortical processing that warrant further investigation in larger samples, although they cannot be considered conclusive. While baseline response patterns differed between participants, both showed increased left parietal activity during tactile stimulation. The inversion of HbO responses between the pre-intervention and intervention phases points to potential sex-related differences in hemodynamic trajectories. Nevertheless, these results remain preliminary, and larger, well-powered studies are required to determine whether these patterns reflect stable, sex-dependent developmental changes.

## 1. Introduction

The first year of life represents one of the most dynamic periods of human brain development. During this stage, rapid neuronal growth, cortical organization, synaptogenesis, myelination, and the establishment of large-scale functional networks support the emergence of sensory, motor, and cognitive abilities. Because of the remarkable neuroplasticity of the infant brain, environmental stimuli experienced during this period may substantially influence brain maturation and subsequent neurodevelopment. Consequently, disturbances occurring during these early stages may have long-lasting effects on neurological function throughout life [1,2].

Among the earliest environmental inputs, tactile stimulation plays a central role in shaping early brain development. Gentle tactile stimulation activates cutaneous mechanoreceptors whose afferent signals ascend through the dorsal column–medial lemniscus pathway toward the thalamus and primary somatosensory cortex, promoting the maturation of sensorimotor networks [3]. Repeated somatosensory experiences during infancy contribute to synaptic refinement, functional connectivity, and neurovascular coupling, processes that are essential for normal motor and cognitive development. Experimental and clinical evidence suggests that early tactile experiences can modulate cortical activity and influence the organization of neural circuits during periods of heightened developmental plasticity [3,4,5,6].

Infant massage performed early in life provides tactile stimulation that can support sensorimotor and cognitive development, facilitating brain maturation [4,7]. Previous studies have demonstrated beneficial effects of massage on neurodevelopmental outcomes, particularly in preterm infants. For example, Procianoy et al. [8] reported improved developmental indices at two years of corrected age following neonatal massage therapy. Likewise, the progressive maturation of cortical networks involving temporal, parietal, and occipital regions during early infancy suggests that sensory stimulation may facilitate the functional organization of these developing circuits [5]. However, the neurophysiological mechanisms underlying these effects remain poorly understood, particularly in healthy term infants.

Brain maturation may also be influenced by biological sex. Prenatal exposure to sex hormones contributes to neural differentiation during fetal development and may influence cortical organization, white and gray matter distribution, and functional connectivity [9,10]. Structural neuroimaging studies have reported sex-related differences in brain growth during the first months of life [2,11,12,13], while studies in very preterm infants suggest greater vulnerability in males regarding functional connectivity and long-term neurodevelopmental outcomes [14]. Likewise, some cortical and subcortical regions, including the amygdala, hippocampus, and temporal areas, appear to follow sex-specific developmental trajectories during early infancy [15]. Nevertheless, little is known about whether these anatomical differences translate into different functional cortical responses to early tactile stimulation.

Most evidence regarding early sex-related brain differences has been obtained using magnetic resonance imaging (MRI), which has substantially improved our understanding of structural brain maturation [1,11,12,13,14]. However, MRI provides limited information regarding real-time cortical responses to sensory stimulation and presents practical limitations when studying awake infants. In contrast, electroencephalography (EEG) offers excellent temporal resolution for evaluating cortical electrical activity, whereas functional near-infrared spectroscopy (fNIRS) provides a non-invasive assessment of cerebral hemodynamic responses. The complementary use of EEG and fNIRS therefore represents a promising approach for investigating the immediate neurophysiological effects of tactile stimulation during early brain development [16,17].

Despite growing evidence supporting the benefits of tactile stimulation during infancy, little is known about the cortical electrical and hemodynamic responses elicited by massage in healthy term infants or whether these responses differ according to sex. Therefore, the present exploratory study aimed to investigate potential sex-related differences in cortical electrical activity and cerebral oxygenation induced by standardized infant massage using the assessment of EEG and fNIRS.

## 2. Materials and Methods

### 2.1. Design and Sample

**Figure 1:**
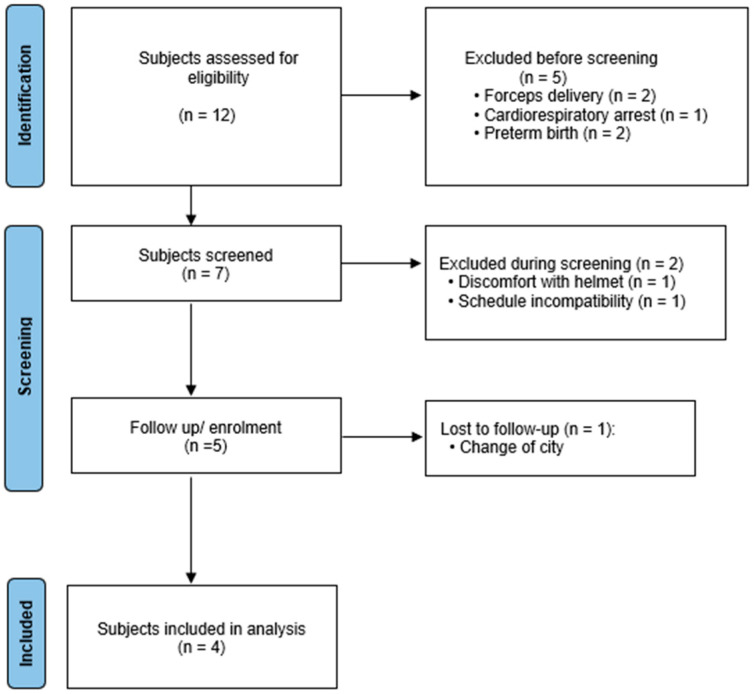
Participant recruitment and study flow diagram.

This single-center prospective exploratory study was conducted over a three-month period. A total of four healthy full-term infants were included in the study, comprising two females and two males. All infants had a corrected age of 11 weeks at the time of assessment and had experienced uncomplicated pregnancies and deliveries. Participant recruitment and selection are summarized in the study flow diagram (Figure 1).

Inclusion criteria:

• Full-term infants (gestational age ≥ 37 weeks).
• Corrected age of 11 weeks.
• Both sexes.
• Absence of congenital or perinatal pathology at birth.

Exclusion criteria:

• Neurological or respiratory disorders, birth complications, including instrumented deliveries (forceps or vacuum-assisted delivery).
• Maternal history of, or ongoing, pharmacological treatment for chronic or acute medical conditions during pregnancy.

The study protocol was approved by the Research Ethics Committee of the University of Salamanca (Code 806), and written informed consent was obtained from the parents or legal guardians of all participants before enrollment. The criteria established by the Declaration of Helsinki were followed. Likewise, in accordance with the Data Protection Law, all the participants’ data were coded, preventing their identification and guaranteeing anonymity.

### 2.2. Experimental Procedure and Outcome Variables

All recordings were performed under standardized environmental conditions in a quiet room. Infants were assessed shortly after feeding while awake, calm, and accompanied by one parent to minimize distress and spontaneous movements. Although behavioral state was monitored continuously by the research team throughout the recording, no formal standardized behavioral state scale was applied [18].

The electrode montage was selected according to the characteristics of the neonatal EEG system used in this study. Because recordings were performed in awake 11-week-old infants, a reduced eight-channel configuration was chosen to minimize preparation time, participant discomfort, and movement-related artifacts. Although this configuration did not include central (C3/C4) or temporal electrodes, it enabled reliable acquisition of cortical electrical activity under natural recording conditions.

Before data acquisition, EEG electrodes and the fNIRS probe were positioned according to the manufacturer’s recommendations.

The experimental protocol consisted of three consecutive phases:

(1) a 5 min resting baseline;
(2) a standardized 5 min infant massage intervention;
(3) a 5 min post-intervention resting period.

The massage intervention followed a standardized protocol and was performed under the supervision of the same experienced physiotherapist throughout the study to ensure consistency in the sequence and execution of the massage maneuvers.

During the massage, the infant remained in the supine position while gentle tactile stimulation was administered following the standardized infant massage protocol previously described by Llamas-Ramos et al. [19]. The intervention included sequential massage of the lower limbs, upper limbs, abdomen and thorax using gentle, rhythmic movements.

Throughout the three phases, cortical electrical activity and cerebral hemodynamic responses were recorded simultaneously.

Cortical electrical activity constituted the primary neurophysiological outcome and was assessed using electroencephalography (EEG). Relative spectral power was calculated for the delta (0.5–4 Hz), theta (4–8 Hz), alpha (8–13 Hz), beta (13–30 Hz), and gamma (30–50 Hz) frequency bands.

Cerebral oxygenation was assessed using functional near-infrared spectroscopy (fNIRS). Changes in oxygenated hemoglobin (HbO_2_) and deoxygenated hemoglobin (HHb) concentrations were used as indicators of cortical hemodynamic activation throughout the experimental protocol.

### 2.3. EEG Acquisition

**Figure 2:**
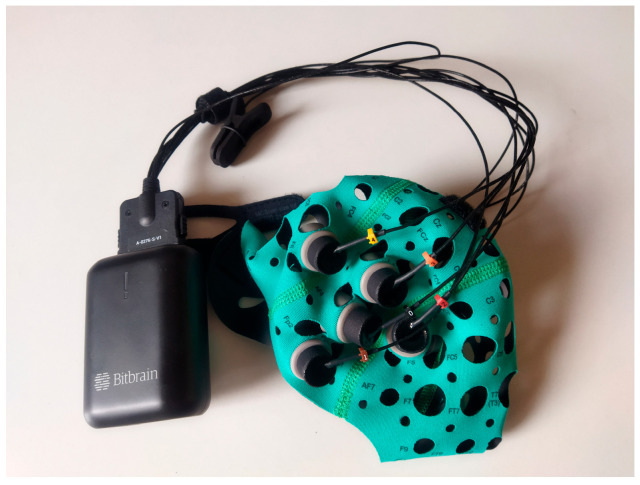
EEG device.

To assess the brain electrical activity, the EEG Versatile 8 (Bit & Brain Technologies S.L., Zaragoza, Spain) was selected. It consists of a cap and 8 electrode channels that collect the cortical electrical activity signals. The electrode distribution followed the “10-5 system” for electrode placement: Fpz, GND, F3, F4, P3, P4, O1 and O2 [20]. (Figure 2).

### 2.4. fNIRS Acquisition

**Figure 3:**
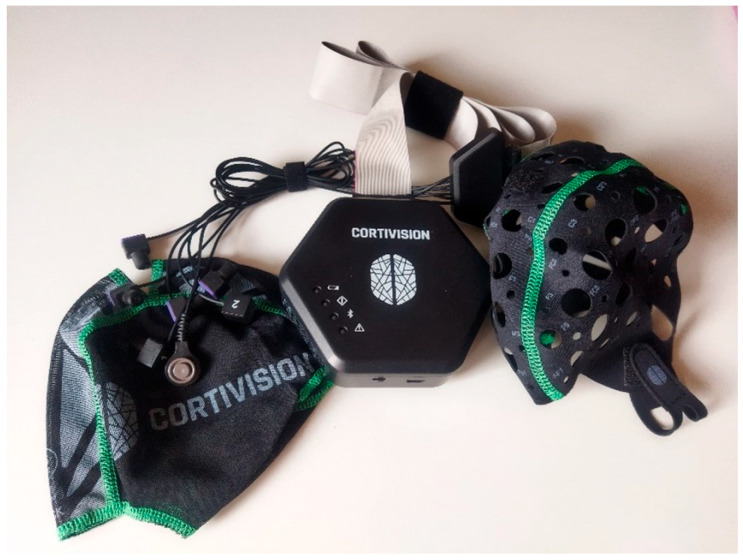
CORTIVISION PHOTONCAP type C20.

CORTIVISION fNIRS technology (CORTIVISION PHOTONCAP type C20-Baby kit, Cortivision Sp. z o.o., Lublin, Poland) was used for the study and measurement of cerebral oxygenation (Figure 3).

The fNIRS probe consisted of four light emitters and two detectors arranged according to the manufacturer’s infant configuration. The source–detector separation was approximately 30 mm, consistent with recommendations for functional cortical measurements in young infants. This configuration was selected to maximize cortical sensitivity while maintaining adequate probe stability and participant comfort during awake recordings [21,22].

### 2.5. Data Processing

A third researcher, blinded to the nature of the subjects given the coding of the data to guarantee anonymity, oversaw analyzing all the data obtained. This researcher was never present during the interventions.

EEG was preprocessed with the Harvard Automated Processing Pipeline for Electroencephalography (HAPPE v4) [23] for developmental EEG data, applying a band-pass filter from 1 to 31 Hz, maintaining the default low-density configuration settings, and segmenting the data into two-second epochs. For awake, sensory-stimulated 11-week-old infants, the 4–30 Hz band targets the location of the functionally relevant signal while excluding the band most compromised by state and artifact. Developmentally, the infant brain shifts power from delta toward faster activity. By 2 months, infants show a theta peak near 5.5 Hz and a transient ~9.5 Hz alpha peak [24], consistent with the maturational redistribution of power from low to high frequencies documented across infancy [25]. This matters because, in neonates, delta is fundamentally a sleep signature (dominant in quiet sleep and tracé alternant) whereas wakefulness elevates faster, attention-related rhythms [26]. Crucially, stimulus-evoked infant responses occur predominantly above the delta band: mu desynchronization during action execution and observation [27], sensorimotor mu and beta during innate rhythmic movement [28], evoked theta to unexpected visual events in 9-month-olds [29], and binding-related gamma to object processing in 8-month-olds [30]. Therefore, 4–30 Hz captures the task-locked activity a stimulation paradigm is designed to elicit. These infant bands have well-established functional correlates: theta with anticipatory and sustained attention [31] and alpha/mu with sensorimotor processing [25]. Finally, awake infants move far more than sleeping neonates, a major source of data attrition in infant EEG/ERP studies [32], and naturalistic infant movement contaminates the record, with the heaviest baseline drift in the sub-4 Hz range [33]. A 3 Hz high-pass removes much of that low-frequency contamination, improving signal-to-noise ratio in the retained bands. In short, for this specific infant population and paradigm, 4–30 Hz is state-appropriate, captures stimulus-evoked and functionally interpretable oscillations, and controls the worst artifacts.

Preprocessing was performed in Matlab v24.1.0.2653294 (R2024a) (Natick, Massachusetts: The MathWorks Inc. Natick, MA, USA). Power spectrum density (PSD) was calculated between 4 Hz and 30 Hz and plotted with EEGLab v2024.1 [34]. Statistical analysis was also performed with EEGLab v2024.1 built-in study parametric functions.

Homer3 (Boston University) [35] was used to preprocess the fNIRS signal in *.snirf format. The preprocessing approach followed the most robust and reliable pipeline for infant fNIRS data identified by Geminagi and Gervain [36]. Specifically, pipeline A was implemented, which included conversion of raw intensity signals to optical density (hmrIntensity2OD), estimation of oxygenated and deoxygenated hemoglobin concentrations (hmrOD2Conc), band-pass filtering with cutoff frequencies of 0.01 Hz (high-pass) and 0.7 Hz (low-pass) using the hmrBandpassFilt function, and correction of motion-related artifacts via the hmrMotionCorrectPCSrecurse function (tMotion = 0.2, tMask = 1.0, StdevThresh = 50, AmpThresh = 0.1). Lastly, hemoglobin concentrations were averaged across each time window using the hmrBlockAvg function. After the preprocessing, the average percentages of corrected signal each interval (pre-rest, intervention, post-rest) were 32%, 7%, and 12%, respectively, in the male, and 2%, 10%, and 24%, respectively, in the female.

AtlasViewer v2.44 [37] was used to project the Optode probes and HbO concentrations into the cortex template of 44 weeks PMA (post-menstruation age), which is the available model closest to the age of the participants in the study (11 weeks gestational age ~48 weeks PMA), which make the optodes project into the primary motor (M1) and somatosensory (S1) cortex bilaterally.

Given the exploratory nature of this pilot study, the fNIRS analyses were intended to characterize temporal patterns of cortical hemodynamic responses during the experimental protocol. Therefore, the statistical findings should be interpreted as exploratory rather than confirmatory and require validation in larger participant-level studies.

## 3. Results

### 3.1. Cortical Activity (EEG)

**Figure 4:**
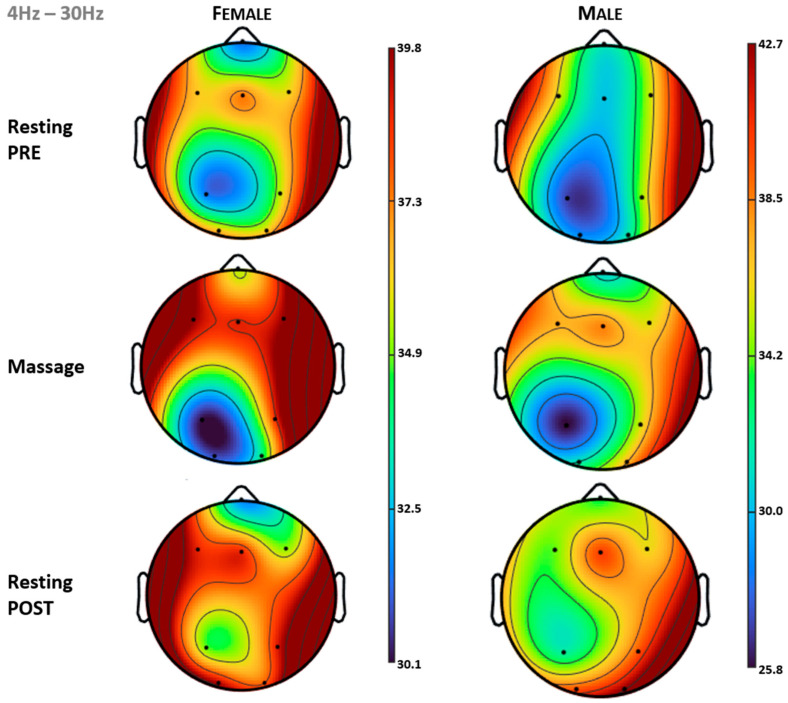
**Top**: Topographical plots of the PSD between 4 Hz and 30 Hz for all subjects and conditions, and electrodes. **Bottom**: Topographical plots of the PSD in the same frequency band for each subject in the study (columns) and each condition (rows).

Figure 4 (top) shows the PSD topography between 4 Hz and 30 Hz for the subjects (rows) across each condition (columns). Relative to the 5 min baseline period, the intervention induced a similar effect in both subjects, with increased PSD in the central frontal area (FCz) that persisted during the post-intervention condition. The intervention also produced a decrease in PSD in the left parietal area (P3) during massage, followed by an increase above baseline in the post-intervention period (Table 1).

### 3.2. Brain Oxygenation (fNIRS)

Because of the exploratory design and the limited number of participants, the fNIRS findings are presented descriptively. The observed HbO changes are intended to characterize temporal response patterns during the massage intervention and post-intervention period rather than to establish confirmatory statistical differences.

**Figure 5:**
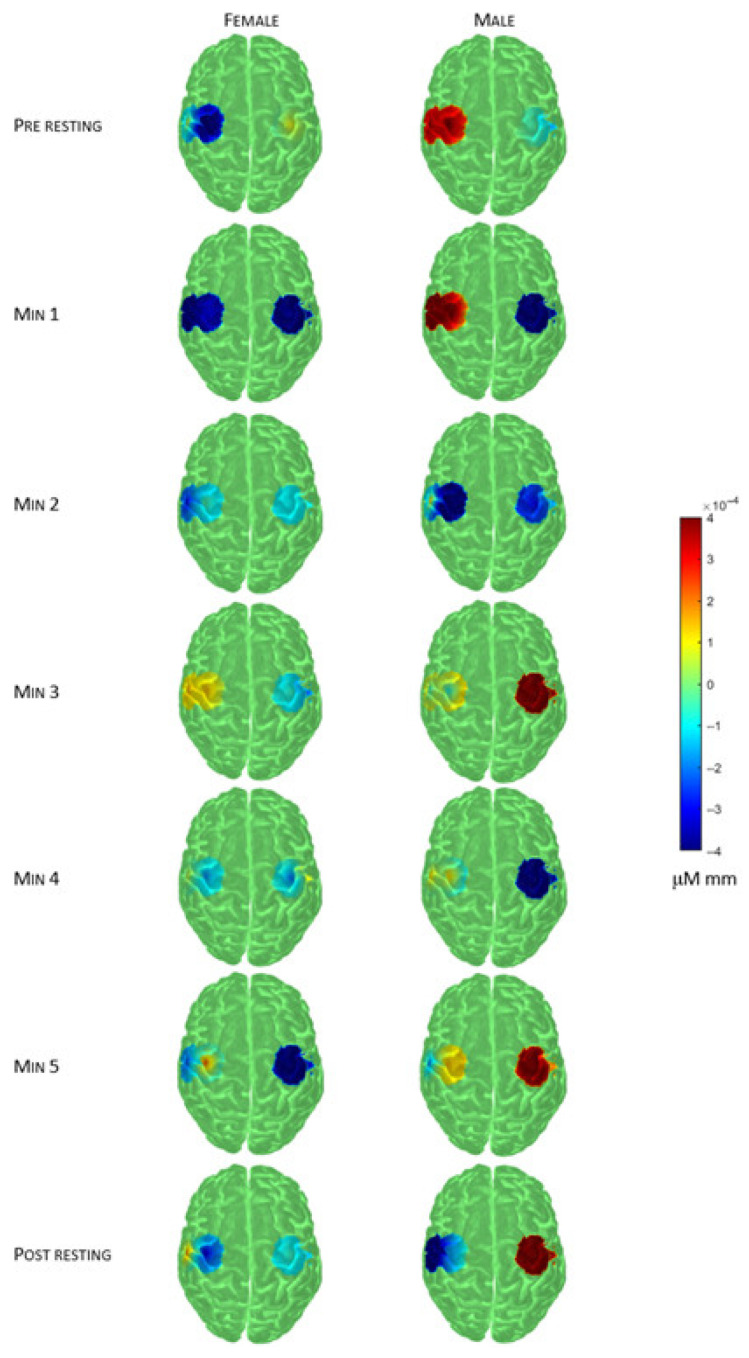
Average oxygenated hemoglobin concentration (HbO) for each time interval (row) and for each sex (columns) projected over standard cortex template. Minutes 1–5 represent the five consecutive one-minute intervals of the massage intervention. The post-massage resting period was analyzed separately.

Figure 5 illustrates sex-specific differences in cortical activity over the motor cortex relative to the 5 min baseline period recorded prior to the intervention. In the female participant, activity decreased bilaterally during the first massage minute of the intervention, then increased bilaterally in the second massage minute, with the increase persisting only in the left hemisphere during the third massage minute. Activity decreased bilaterally in the fourth massage minute, followed by a decrease in the right hemisphere and an increase in the dorsal left hemisphere during the fifth massage minute. In the post-intervention resting period, activity increased in the right hemisphere and in the most ventral part of the left hemisphere. In the male participant, activity decreased in the right hemisphere during the first massage minute, then decreased bilaterally in the second massage minute. During the third massage minute, activity increased bilaterally, more prominently in the right hemisphere. In the fourth massage minute, activity decreased in the right hemisphere, and in the fifth massage minute it increased bilaterally, again more pronouncedly in the right hemisphere. Post-intervention, activity decreased in the left hemisphere only.

All participants showed similar HbO concentration patterns during the second minute of intervention. Overall, the male participant exhibited higher activation throughout the intervention, mainly in the right hemisphere, which remained activated during the post-intervention resting period relative to baseline (Table 2).

In contrast, the female participant exhibited greater activation in the left hemisphere during the intervention, which was partially maintained in the ventral region during the post-intervention resting period relative to the 5 min baseline period. During the pre-intervention resting period, HbO patterns were inverted between the participants, with the male showing higher activation in the left hemisphere, while the female showed higher activation in the right hemisphere. Overall, the effects of the intervention relative to the baseline (pre-intervention) differed between sexes.

**Table 2:** Mean (standard deviation) values of oxygenated hemoglobin concentration (HbO), normalized to the pre-intervention resting periods (z-scores), are presented for each optode across time intervals (rows) and by sex (columns).

Optode	Time Interval	Female	Male
Left superior (1-1)	Massage Min 1	2.156 (1.479)	0.386 (1.696)
	Massage Min 2	3.577 (0.814)	−5.465 (2.446)
	Massage Min 3	4.618 (1.032)	−2.326 (1.812)
	Massage Min 4	2.687 (0.841)	−1.332 (2.299) ^a^
	Massage Min 5	5.112 (1.006)	−1.241 (2.056) ^a^
	Post rest	1.752 (0.693)	−2.636 (1.065)
Left inferior (2-1)	Massage Min 1	−2.369 (1.596)	2.887 (1.646)
	Massage Min 2	−1.782 (0.843)	−1.742 (0.843)
	Massage Min 3	0.438 (1.436)	−3.211 (1.064)
	Massage Min 4	−0.069 (1.013)	−1.934 (1.256)
	Massage Min 5	−1.453 (1.645)	−4.402 (1.149)
	Post rest	0.645 (0.505)	−9.580 (3.300)
Right superior (3-2)	Massage Min 1	−1.173 (0.706) ^a^	−10.909 (6.910)
	Massage Min 2	−0.520 (0.527) ^b, c, d^	−2.187 (8.217)
	Massage Min 3	−0.397 (0.766) ^b, e, f^	8.831 (4.970)
	Massage Min 4	−0.851 (0.490) ^a, c, e, g^	−8.118 (5.467)
	Massage Min 5	−1.771 (0.728)	5.572 (6.660)
	Post rest	−0.389 (0.357) ^d, f, g^	11.395 (6.903)
Right inferior (4-2)	Massage Min 1	−2.515 (1.521)	−0.031 (1.368)
	Massage Min 2	−0.511 (0.782)	1.382 (2.375)
	Massage Min 3	−1.834 (0.690)	2.423 (0.523)
	Massage Min 4	0.790 (1.100)	0.676 (0.780)
	Massage Min 5	−1.616 (1.021)	1.527 (0.754)
	Post rest	−1.012 (0.606)	2.110 (0.712)

The female participant showed a slight decrease in activation in the right hemisphere and a modest increase in the most ventral region of the left hemisphere relative to the 5 min baseline period. In contrast, the male participant displayed the opposite pattern, with a marked increase in the right hemisphere and a pronounced decrease in the left hemisphere.

**Figure 6:**
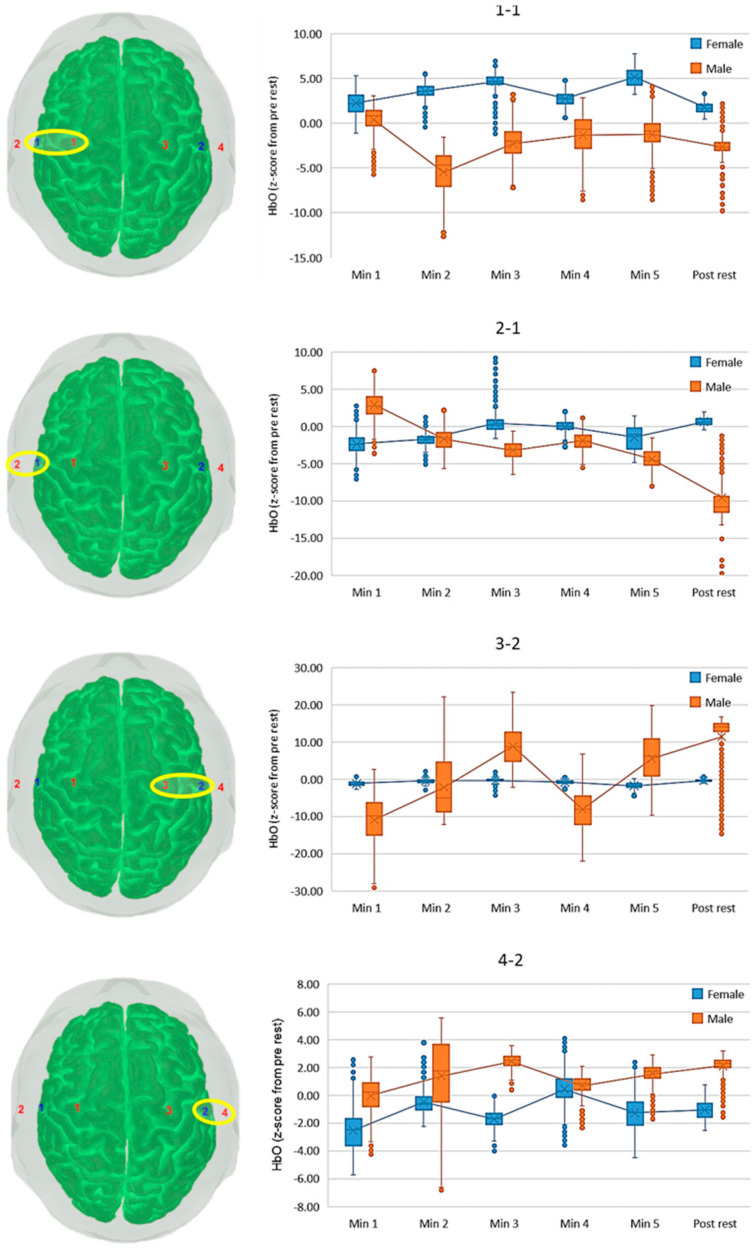
Box plots of the oxygenated hemoglobin concentration (HbO) time points normalized with respect to the pre-intervention resting period (z-scores) for each time interval (*x*-axis), each sex (colors), and each pair of detector-emitter optodes (rows).

Figure 6 illustrates the changes in activation relative to the 5 min baseline period for each participant and targeted motor cortical region. For the most rostral area of the left hemisphere (optode 1-1), the female subject increased the activation during the intervention and decreased during the post-intervention period. The male subject, however, kept the activation during minute 1 of the intervention and then drastically decreased it in the second minute. From then, the activation increased and remained below the baseline/pre-intervention phase.

In the most ventral left area (2-1 optode) the activity increased during the first minute of intervention for the female subject but then decreased progressively during the remaining time intervals (Figure 6). The male subject, however, started with a decrease during the first minute but then monotonously increased the activity to values comparable to the pre-intervention period until the third minute, and then kept that activity until the end of the session.

At the right superior optode (3-2) the activity was nearly constant for the female subject. However, the male subject presented high oscillations, starting with a high decrease in the first minute, then substantially increasing until the third minute, with a subsequent decrease again in the fourth minute and a final increase that kept the activity higher than the one in the pre-intervention period.

Finally, in optode 4-2 (right inferior), relative to the 5 min baseline period, both participants showed a similar overall time pattern, with a progressive increase in activation despite minor oscillations (Figure 6). The male subject exhibited higher activation than baseline/pre-intervention period throughout the session, whereas the female participants showed lower activation across the session, primarily due to a decrease during the first minute.

## 4. Discussion

The present exploratory study investigated the effects of standardized infant massage on cortical electrical activity and cerebral oxygenation in healthy term infants using the assessment of electroencephalography (EEG) and functional near-infrared spectroscopy (fNIRS). Overall, the findings demonstrate that tactile stimulation is associated with measurable neurophysiological changes during early infancy, characterized by transient modulation of cortical electrical activity together with dynamic hemodynamic responses. Although exploratory sex-related differences were observed, the principal finding of this study is that structured tactile stimulation elicited reproducible functional brain responses during a period of rapid neurodevelopment. Therefore, although massage appears to induce measurable neurophysiological responses, the potential influence of spontaneous variations in infant behavioral state cannot be completely excluded. Given the inclusion of only four participants (two males and two females), any sex-related differences should be interpreted with considerable caution and regarded as preliminary observations requiring confirmation in adequately powered studies.

Touch is one of the earliest and most relevant sensory experiences after birth and plays a fundamental role in shaping sensorimotor development. Gentle tactile stimulation activates cutaneous mechanoreceptors and ascending somatosensory pathways, promoting activity-dependent cortical plasticity during a period of intense synaptogenesis and functional network maturation [4,5]. Beyond its discriminative function, tactile stimulation also engages affective touch pathways that contribute to early socio-emotional and sensorimotor development [38]. Functional neuroimaging studies have demonstrated that healthy term infants already exhibit organized resting-state networks involving sensory, parietal, temporal, and prefrontal cortical regions, providing the neurodevelopmental substrate through which tactile stimulation may modulate early brain function [39]. In agreement with previous studies demonstrating beneficial effects of infant massage on neurodevelopment [8], the present results suggest that massage may induce measurable changes in both cortical electrical activity and cerebral oxygenation. These observations support the hypothesis that tactile stimulation can contribute to early functional brain organization and can reinforce the value of non-invasive sensory interventions during infancy.

The EEG recordings revealed a transient reduction in cortical electrical activity within the left parietal region during massage, followed by increased activity during the post-intervention resting period compared with baseline. Rather than indicating reduced cortical function, this pattern may reflect adaptive modulation of neuronal activity during continuous tactile stimulation followed by post-stimulation cortical reorganization. Similar transient changes in cortical oscillatory activity have been described following somatosensory stimulation, supporting the concept of experience-dependent neuroplasticity during early development. Although the present exploratory design does not allow causal inferences, these findings demonstrate that EEG is sufficiently sensitive to detect functional cortical responses to massage in very young infants.

The fNIRS recordings complemented these findings by demonstrating dynamic changes in cerebral oxygenation throughout the intervention. Variations in oxygenated hemoglobin (HbO) are considered reliable indicators of neurovascular coupling and neuronal activation [40,41]. During the second minute of massage, HbO concentrations were comparable between male and female infants, whereas subsequent time intervals showed progressive changes in cortical oxygenation. These hemodynamic responses are consistent with the physiological relationship between neuronal activation and regional cerebral blood flow and support the complementary use of EEG and fNIRS for studying functional brain responses during infancy.

Although the primary objective of this study was to characterize the cortical response to tactile stimulation, exploratory analyses suggested potential differences between male and female infants. Baseline cortical activation patterns differed between sexes, and distinct hemispheric activation profiles were observed during massage and the post-intervention period. Male infants exhibited relatively greater activation within the right hemisphere, whereas female infants showed greater activation within the left hemisphere. While these observations should be interpreted cautiously because of the limited sample size, they are consistent with previous neuroimaging studies suggesting sex-related differences in early brain organization [42,43].

Evidence regarding sex differences in cortical responses to tactile stimulation during early infancy remains scarce. Most studies investigating affective or social touch have focused on adults or older children, and sex-specific cortical responses during early infancy remain poorly characterized [44,45,46]. Most previous investigations have focused on language processing, social interaction, visual perception, sleep, or cognitive development [47,48,49,50,51,52], reporting sex-dependent patterns of cortical activation during the first years of life. Girls frequently demonstrate greater left-hemisphere activation during language-related tasks, whereas boys often exhibit more bilateral or right-lateralized activation [41,53,54]. Conversely, studies evaluating painful tactile stimulation have generally reported similar cortical activation patterns between sexes [55]. Considering that infants younger than three months rely predominantly on somatosensory information to interact with their environment [56], the present findings provide preliminary evidence that massage-induced tactile stimulation may also elicit differential functional responses according to sex. Nevertheless, these observations should be considered hypothesis-generating and require confirmation in larger cohorts. Previous EEG studies in adults have also reported sex-related differences in cortical oscillatory activity and auditory processing, although neuroimaging findings remain inconsistent [57,58].

One of the strengths of the present study is the application of EEG and fNIRS, which provides complementary information regarding neuronal electrical activity and cerebral hemodynamics. Although magnetic resonance imaging has substantially improved our understanding of structural brain development [43], its practical limitations in awake infants restrict its usefulness for studying dynamic sensory responses [22,59,60,61]. In contrast, EEG offers excellent temporal resolution, whereas fNIRS allows continuous, non-invasive monitoring of cerebral oxygenation in naturalistic experimental conditions. Nevertheless, comparison with previous studies remains difficult because of differences in instrumentation, optode configuration, recording duration, and cortical coverage [34,41,62]. In the present study, the reduced number of optodes was selected to accommodate the small head size of participants while minimizing discomfort and movement-related artifacts.

Several limitations should be acknowledged. First, the sample size was intentionally small because this study was designed as an exploratory pilot investigation. Consequently, statistical power was limited, particularly for the analysis of sex-related differences, which should therefore be interpreted cautiously. Second, cortical recordings were restricted to somatosensory regions, precluding evaluation of whole-brain functional responses. Because no short-separation channels were available, superficial scalp hemodynamics cannot be completely excluded from the recorded fNIRS signals. Third, although EEG and fNIRS recordings were obtained under standardized experimental conditions, including assessment after feeding while infants were awake and calm, no validated behavioral state scale was used to objectively document sleep–wake status throughout the recording [18]. Consequently, differences in behavioral state cannot be completely excluded as a potential contributor to the observed EEG and fNIRS responses. Furthermore, because of the exploratory nature of this pilot study and the small sample size, the fNIRS findings should be interpreted as descriptive observations intended to generate hypotheses for future research rather than as confirmatory evidence. Fourth, an additional limitation is the restricted EEG montage. The absence of central electrodes (C3/C4) precluded direct assessment of the primary somatosensory cortex, which is expected to play a key role in tactile processing. Fifth, although the massage protocol was standardized and supervised by the same physiotherapist throughout the study, no objective assessment of protocol fidelity (e.g., stroke pressure, movement velocity, or video-based verification) was performed. Future studies should incorporate quantitative fidelity measures to further improve reproducibility. More investigations using high-density EEG systems should incorporate central and temporal electrodes to improve spatial characterization of massage-induced cortical responses. Future studies should include larger and more diverse populations, longitudinal follow-up, broader cortical coverage, standardized behavioral assessment, and different types of sensory stimulation to better characterize the mechanisms underlying tactile-induced neuroplasticity.

Despite these limitations, the present study demonstrates the feasibility of recording EEG and fNIRS during standardized infant massage, providing preliminary evidence that multimodal neurophysiological monitoring may be useful for investigating early sensory processing during infancy.

Future confirmatory studies should be designed as adequately powered, prospective investigations including substantially larger samples of healthy term infants, with balanced representation of both sexes to allow formal comparisons. Although this exploratory pilot study was not intended to estimate effect sizes for sample size calculations, the variability observed in both EEG and fNIRS measures provides useful information for planning future power analyses. A longitudinal design with repeated assessments across early infancy would help determine whether the neurophysiological responses observed here represent stable developmental trajectories or transient maturational phenomena. Future studies should also incorporate standardized behavioral state assessment, broader cortical coverage with high-density EEG and expanded fNIRS montages, and predefined primary outcomes to enable robust confirmatory statistical analyses. Such studies would provide the evidence necessary to determine whether the preliminary sex-related patterns identified in the present pilot study reflect true biological differences in early cortical responses to tactile stimulation.

## 5. Conclusions

In conclusion, the present findings suggest that standardized infant massage is a safe, feasible, and non-invasive intervention capable of eliciting measurable changes in cortical electrical activity and cerebral oxygenation during early infancy. Although these preliminary observations require confirmation in larger longitudinal studies, they support the potential role of tactile stimulation as an adjunct strategy to promote early brain maturation and highlight the usefulness of EEG and fNIRS to investigate functional brain development during the first months of life.

## Figures and Tables

**Table 1:** Average (standard deviation) values of the normalized (10*log) PSD in the P3 electrode for each subject (rows) and each condition (columns).

	10*log (PSD) in P3		
Subject	Pre	Massage	Post
Female	−4.566 (−1.251)	−6.726 (−4.075)	−1.611 (1.624)
Male	−9.758 (−7.206)	−12.594 (−10.741)	−0.948 (3.555)

## Data Availability

The data sets generated during and/or analyzed during the current study are available from the corresponding author on request.

## Abbreviations

The following abbreviations are used in this manuscript:

EEG	Electroencephalography
fMRI	Functional magnetic resonance imaging
fNIRS	Functional near-infrared spectroscopy
HbO	Oxygenated Hemoglobin
HbR	Deoxygenated Hemoglobin
Hz	Herzio
MRI	Magnetic Resonance Imaging
PSD	Power spectrum density
